# Cost-effectiveness of adjuvant chemotherapy for curatively resected gastric cancer with S-1

**DOI:** 10.1186/1471-2407-13-443

**Published:** 2013-10-01

**Authors:** Akinori Hisashige, Mitsuru Sasako, Toshifusa Nakajima

**Affiliations:** 1 The Institute of Healthcare Technology Assessment, 2-24-10, Shomachi, 770-0044, Tokushima, Japan; 2 Department of Upper Gastrointestinal Surgery, Hyogo College of Medicine, 663-8501, Hyogo, Japan; 3 Division of Surgery, The Cancer Institute Hospital, 135-8550, Tokyo, Japan

**Keywords:** Chemotherapy, S-1, Adjuvant therapy, Gastric cancer, Cost-effectiveness, Quality-adjusted life-year

## Abstract

**Background:**

The effectiveness of specific regimens of adjuvant therapy for gastric cancer has not been verified by large clinical trials. Recently, several large trials attempted to verify the effectiveness of adjuvant therapy. The Adjuvant Chemotherapy Trial of TS-1 for Gastric Cancer in Japan, a randomized controlled trial of adjuvant S-1 therapy for resected gastric cancer, demonstrated significant improvement in overall and relapse-free survival, compared to surgery alone. To evaluate value for money of S-1 therapy, cost-effective analysis was carried out.

**Methods:**

The analysis was carried out from a payer’s perspective. As an economic measure, cost per quality-adjusted life-year (QALY) gained was estimated. Overall survival was estimated by the Kaplan-Meier method, up to 5-year observation. Beyond this period, it was simulated by the modified Boag model. Utility score is derived from interviews with sampled patients using a time trade-off method. Costs were estimated from trial data during observation, while in the period beyond observation they were estimated using simulation results. To explore uncertainty of the results, qualitative and stochastic sensitivity analyses were done.

**Results:**

Adjuvant S-1 therapy gained 1.24 QALYs per patient and increased costs by $3,722 per patient for over lifetime (3% discount rate for both effect and costs). The incremental cost-effectiveness ratio (95% confidence intervals) for over lifetime was estimated to be $3,016 ($1,441, $8,840) per QALY. The sensitivity analyses showed the robustness of these results.

**Conclusion:**

Adjuvant S-1 therapy for curatively resected gastric cancer is likely cost-effective. This therapy can be accepted for wide use in Japan.

## Background

Gastric cancer is a major health problem worldwide. It ranks second in all causes of death from cancer, with about 700,000 confirmed deaths annually [1,2]. In Japan, although its mortality ranks also second and has decreased in recent years, it still has the highest incidence despite advances in prevention and treatment [3]. While the internationally accepted standard treatment for patients with potentially resectable disease was surgery alone [4,5], meta-analyses of adjuvant chemotherapy for gastric cancer during the last few decades have shown reductions in mortality up to 18% [6,7]. However, these reductions were considered insufficient to change clinical practice.

Recently, the effectiveness of specific regimens for resectable gastric and/or gastroesophageal cancer has been verified in large clinical trials. The chemoradiation therapy (INT-0116) in the US in 2001 [8], the perioperative chemotherapy (MAGIC) in Europe in 2006 [9], and the postoperative chemotherapy (ACTS-GC) in Japan in 2007 [10,11] improved significantly overall survival (OS), and relapse-free survival (RFS) or progression-free survival (PFS), compared to surgery alone.

These studies have led to a new phase in the treatment of gastric cancer, even though there are several issues under discussion concerning them [5,12,13]. Postoperative chemoradiotherapy, perioperative triplet-chemotherapy, and postoperative S-1 mono-chemotherapy are now the standard therapies in the US, Europe and Japan, respectively [5,12]. Also, the status of adjuvant treatment of gastric cancer has been evolving to improve and optimize the current standard of care across national boundaries.

Under these circumstances, from a perspective of healthcare policy, in choosing the best treatment among the different options available, clinical benefits of treatments should be balanced against the effects on costs, since rapid growth in healthcare expenditures creates an unsustainable burden. However, economic evaluation of adjuvant therapy for gastric cancer has been greatly lacking.

Our objective was to estimate the cost-effectiveness of adjuvant S-1 therapy in Japan. This study would provide basic information on the cost-effectiveness of adjuvant therapy for gastric cancer in Japan.

## Methods

### Analytical overview

Economic analysis was conducted retrospectively based on the ACTS-GC (ClinicalTrials.gov number, NCT00152217) [10,11]. Patients with completely resected stage II/III gastric cancer, who underwent gastrectomy with extended (D2) lymph-node dissection, were randomly assigned to either oral S-1 (40 mg/m^2^ per day) for 1 year after surgery (n = 529) or surgery alone (n = 530). S-1 is an orally active combination of tegafur, gimeracil, and ostracil in a molar ratio of 1:0.4:1.

As a type of economic analysis [14], a cost-effective analysis was performed. Incremental costs and effectiveness of adjuvant S-1 therapy compared to surgery alone were evaluated. According to the effectiveness measure used (i.e., life-years (LYs) gained and quality-adjusted life-years (QALYs) gained), incremental cost-effectiveness ratios (ICERs) were calculated. In addition, confidence intervals of ICER were also estimated using the non-parametric bootstrap method [14].

The payer of National Health Insurance in Japan was adopted as a perspective of economic analysis [14]. Therefore, for costs, direct medical care costs (e.g., costs of tests, drugs, health care personnel, etc.) were examined, whereas indirect costs (e.g., time costs or production loss among patients and their families) were not considered. As a time horizon for evaluation, three levels of time periods (i.e., observational period [5 years], 10-year follow-up and over lifetime) were considered. As the base case analysis, over lifetime was used, since this period covered long-term consequences of treatment on health and costs.

### Effectiveness

The results of the ACTS-GC were used as evidence of effectiveness in the economic analysis. The clinical results have been presented in detail elsewhere [10,11]. As is shown in Table 1, between the S-1 therapy group and the surgery alone group, no statistical differences were observed in age, sex, pathological tumor stage, or type of lymph-node dissection and gastrectomy. The incidence of adverse events more than grade 3 in the S-1 therapy group was significantly higher than that in the surgery alone group. The OS and RFS rates in the S-1 therapy group were significantly higher than those in the surgery alone group [10,11].

**Table 1:** Characteristics of subjects and clinical outcomes

	**S-1 therapy**	**Surgery alone**
Number of patients	529	530
Age (median)	63	63
Sex (male)	367	369
Cancer stage (TNM classification)		
IB	1	0
II	264	282
IIIA	170	157
IIIB	54	56
IV	40	35
Type of lymph –node dissection		
D1	0	1
D2	501	497
D3	28	32
Type of gastrectomy		
Total	220	201
Distal	301	316
Proximal	4	11
Other	4	2
Adverse events more than grade 3*	155	80
Total no. of relapses	162	221
	%	%
5-year survival (95% CI)	72 (68–76)	61 (57–65)
5-year relapse-free survival (95% CI)	65 (61–70)	53 (49–57)

The results are presented according to ITT (intention to treat): * The results from the safety analysis.

**Figure 1:**
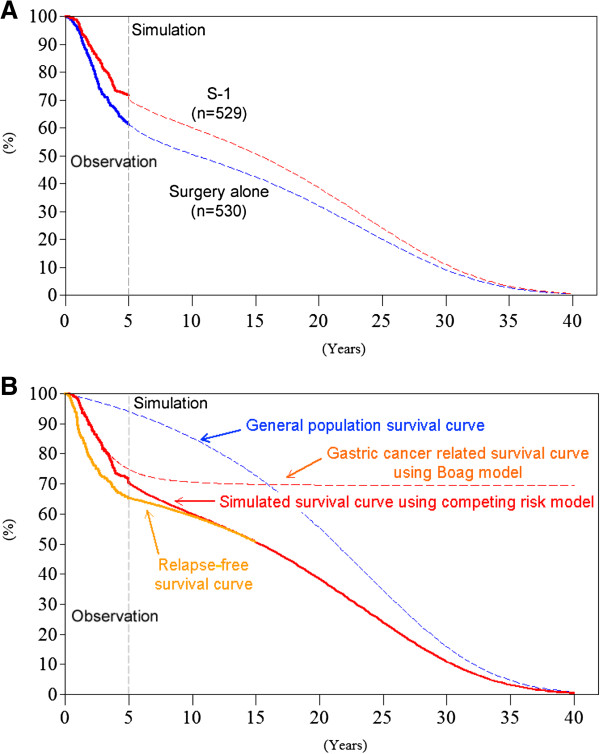
**Survival curve and extrapolated survival estimate. (A)** Survival curve in the S-1 and the control groups, **(B)** Survival curves using Boag and competing risk models and relapse-free survival curve in the S-1 group.

Using patients’ data, OS and RFS were estimated by the Kaplan-Meier method, up to 5 years from randomization. Beyond the observation period of 5 years, OS was simulated using the Boag model [15] combined with the independent competing risk model [16,17] (Figure 1). While there is no explicit standard for extrapolation beyond the observation [18], this model showed an extreme goodness of fit, validated by observational data [17].

In this model, OS curve was decomposed into two components: the disease-specific survival curve and the disease-independent survival curve. In the first curve, only disease-specific (i.e., gastric cancer) deaths were counted as events, and all other deaths were censored; the converse applies to the second curve. The disease-specific survival curve was then fitted by the Boag parametric model. As death from disease becomes rarer with increasing time, the disease-related survival curve approximates to a plateau (Figure 1B, gastric cancer related survival curve using the Boag model).

Instead of the original log-normal model, the log-logistic model was adopted in this analysis, according to the analysis of observational data of this trial. This log-logistic model was also supported by the analysis of a large database for gastric cancer in Japan [19]. In selecting a model among log-logistic, log-normal and Weibull models, Akaike’s Information Criteria (AIC) were used [20].

The second curve, disease-independent curve was simulated by the survival curve of the general population matched for age and sex of the subjects, using national life tables (Figure 1B, general population survival curve).

The two simulated curves were then extended over lifetime and were recombined (multiplied) into a complete overall survival curve, using the competing risk model (Figure 1B, simulated survival curve using competing risk model). Under the competing risk model, the simulated survival rate is simply derived from multiplying the disease-related survival rate by the disease-independent survival rate. The life years were estimated as the area under the curve (AUC). The survival rate and variance were obtained by maximum likelihood estimation of the Boag parameters (i.e., the cure rate, the mean and standard deviation of log survival time). A detailed description of QALY calculation is presented in Appendix.

For RFS, the log-logistic model was also adopted, according to the analysis of observational data in the study [10,11] and AIC (Figure 1B, relapse-free survival curve).

The mean number of LYs and relapse-free LYs for patients in each group was estimated as the area under the OS and RFS curves, respectively [21]. In addition, QALYs were calculated from OS and RFS by weighting each survival in each interval by a utility value for each possible important health state (i.e., remission after surgery and relapse). Utility values for these health states were derived from an interview with random samples of patients in remission after surgery (n = 23) and consecutive patients with relapse (n = 21), with informed consent, by using a time trade-off method. No statistical difference was observed in key characteristics between these samples and the population subjects [10,11]. The mean (and S.D.) of the utility values for remission after surgery and for metastasis were 0.851 (0.121), and 0.349 (0.208), respectively. When the risk of relapse has diminished, the change in utility value for remission after surgery would be considered to be the same as that of the general population. We applied the weighting by age for each year of follow-up, based on a population survey for quality of life in Japan [22].

The utility reduction associated with adverse events was adjusted through the method adopted by Aballea, et al [23]. The utilities for hospitalization and the adverse events with grade 4 were reduced by 50%. Also, 23%, 19% and 36% reduction were applied for nausea, vomiting and stomatitis, and diarrhea, respectively.

### Cost

Costs incurred for resources used during trial and subsequent follow-up were estimated from trial data and their extrapolation. Resource utilization during trial and follow-up was derived from individual patient history data. Since observations on many patients are censored in a clinical trial, subsequent costs are unknown. To correct for censoring, the inverse probability weighting method [21] was applied during the observation period. Beyond the observation period, costs related to gastric cancer (i.e., those for recurrence and end-of-life) were estimated using the simulation results. Costs were estimated from the National Health Insurance perspective using the National Health Insurance reimbursement list and drug price for 2007 [24,25]. The costs of adverse events and a recurrence were estimated based on patients’ records during observation. The chemotherapy for the majority of recurrence was implemented according to the first-line therapy in the Japanese guidelines [26].

As most health economic guidelines (e.g., the UK, Canada, Netherlands, Germany and the US) indicated, unrelated health care costs in the later years of life were not included in this analysis [14]. All costs were converted from Japanese yen to US dollars based on OECD purchasing power parity in 2007 ($1 = \120) [27].

### Discount

Discounting for the time value of money was applied to both costs and effectiveness. In the base case analysis, both costs and effectiveness accruing beyond 1 year were discounted to present values at a rate of 3%, following the recommendations of the US Panel on Cost-Effectiveness in Health and Medicine [28]. However, currently, much debate still surrounds two major points: the underlying discounting model and the differential discount rate for health and cost [28-30]. Therefore, the impact of discounting on the results was examined extensively by sensitivity analysis.

### Sensitivity analysis

The uncertainty of the results was explored by stochastic and qualitative sensitivity analyses of important factors [14,31,32]. The impact of uncertainty on the estimated ICER due to the stochastic nature of sampled data was analyzed by applying a non-parametric bootstrap re-sampling technique (i.e., 5000 times) to both costs and effectiveness. Also, cost-effectiveness acceptability curve (CEAC) and net monetary benefit (NMB) analyses [31,32] were performed. A number of qualitative one-way and two-way sensitivity analyses were conducted to explore the impact of alternative parametric assumptions on the results. These included alternative assumptions concerning time horizon, key cost parameter, recurrence rate, utility value, discount rate and simulation method. Also, the exclusion of end-of-life costs due to gastric cancer was examined by a sensitivity analysis, under the assumption that they may be considered as unrelated healthcare costs.

## Results

### Effectiveness

The mean QALYs (3% discount rate) in each group are shown in Table 2. For 5-year observation, 10-year follow-up and over lifetime, the mean QALYs per patient for adjuvant S-1 therapy were 3.11, 5.08 and 8.65, respectively. Those for surgery alone were 2.84, 4.45 and 7.41, respectively. Adjuvant S-1 therapy gained 0.27, 0.64 and 1.24 QALYs per patient, for each period, respectively (p < 0.05). The difference in QALYs was relatively smaller than that in LYs for 10-year follow-up and over lifetime.

**Table 2:** **Incremental effectiveness and costs of adjuvant S-1 therapy (discount rate: 3**% **for both effectiveness and costs)**

**Period**	**S-1 therapy**	**Surgery alone**	**Incremental effectiveness and costs (95% CI)**
Effectiveness			
	QALYs		
5-year observation	3.11	2.84	0.27 (0.11 – 0.42)
10-year follow-up	5.08	4.45	0.64 (0.28 – 0.99)
Over lifetime	8.65	7.41	1.24 (0.48 – 1.96)
	Costs ($)		
5-year observation	10,802	7,408	3,389 (2,616 – 4,174)
10-year follow-up	12,110	8,523	3,585 (2,750 – 4,411)
Over lifetime	13,057	9,346	3,722 (2,911 – 4,512)
Incremental cost-effectiveness ratio			
	Cost ($) per QALY gained		(95% CI)
5-year observation	12,716		(6,428 – 34,018)
10-year follow-up	5,608		(2,855 – 14,569)
Over lifetime	3,016		(1,441 – 8,840)

CI = confidence interval; QALYs = quality-adjusted life-years.

### Cost

The mean costs (no discounting) per patient in each group for the 5-year observation are shown in Table 3. The mean total cost per patient was $11,103 in the S-1 therapy group, and $7,761 in the surgery alone group. The costs of recurrence and end-of-life were the major component in both groups. Although S-1 therapy added over $4,000 per patient to the ingredient cost of surgery alone, this was partly offset by the reduction of costs in recurrence and end-of-life of gastric cancer. As is shown in Table 2, for 5-year observation, 10-year follow-up and over lifetime, adjuvant S-1 therapy increased costs (3% discount rate) per patient by $3,389, $3,585 and $3,722 respectively, compared to surgery alone (p < 0.05).

**Table 3:** Mean costs per patient during observation period (no discounting)

**Item**	**S-1 therapy**		**Surgery alone**	
**Unit cost ($)**	**Quantity**	**Cost ($)**	**Quantity**	**Cost ($)**
	**(No. of units)**		**(No. of units)**	
Consultation				
Outpatient: 5.8	22.4	131	8.3	49
Treatment				
S-1 drug (mg): 0.3	15,156	4,367	NA	
Prescription: 4.9	10.5	52	NA	
Tests				
Imaging tests				
CT: 124.3	5.1	629	4.9	608
Chest X-ray: 21.1	2.0	42	2.0	43
Echogram: 44.2	1.8	80	1.9	85
Endoscopy: 95.0	1.5	147	1.7	164
Others: 156.0	0.2	34	0.2	38
Laboratory tests				
Blood test: 35.0	21.3	746	9.1	319
Tumor markers: 33.3	8.6	286	8.6	288
Adverse effects				
Anti-ulcerants: 6.0	0.3	2	0.2	1
Anti-biotics: 15.4	0.2	3	0.1	1
Anti-diarrhoeals: 5.4	0.2	1	0.1	0
Anti-emetics: 64.8	0.1	9	0.0	3
G-CSF: 106.9	0.0	3	0.0	0
Blood transfusion: 188.5	0.0	0	0.0	1
Recurrence				
S-1: 3,694	0.1	487	0.3	1,394
Paclitaxel: 5,298	0.1	422	0.0	101
S-1 + cisplatin: 5,594	0.0	227	0.1	244
S-1 + paclitaxel: 5,247	0.0	162	0.0	129
5FU + methotrexate: 4,748	0.0	65	0.0	144
Irinotecan + cisplatin: 5,197	0.0	162	0.0	43
Others: 4,892	0.0	211	0.0	201
End of life				
Drugs/injections	0.3	1,064	0.4	1,367
Treatments	0.3	233	0.4	321
Operations/anesthesia	0.3	213	0.4	296
Diagnostic tests	0.3	221	0.4	353
Total costs per patient	11,103		7,761	
(SD)	(6,832)		(6,787)	

NA = not applicable.

### Incremental cost-effectiveness ratio

As is shown in Table 2, as the base case, the ICER (95% confidence intervals) for over lifetime was estimated to be $3,016 ($1,441, $8,840) per QALY, using the bootstrap method (3% discount rate for both effect and cost). Those for 5-year observation and 10-year follow-up were $12,716 and $5,608 per QALY, respectively. There is little difference between costs per LY gained and costs per QALY gained.

### Sensitivity analysis

**Figure 2:**
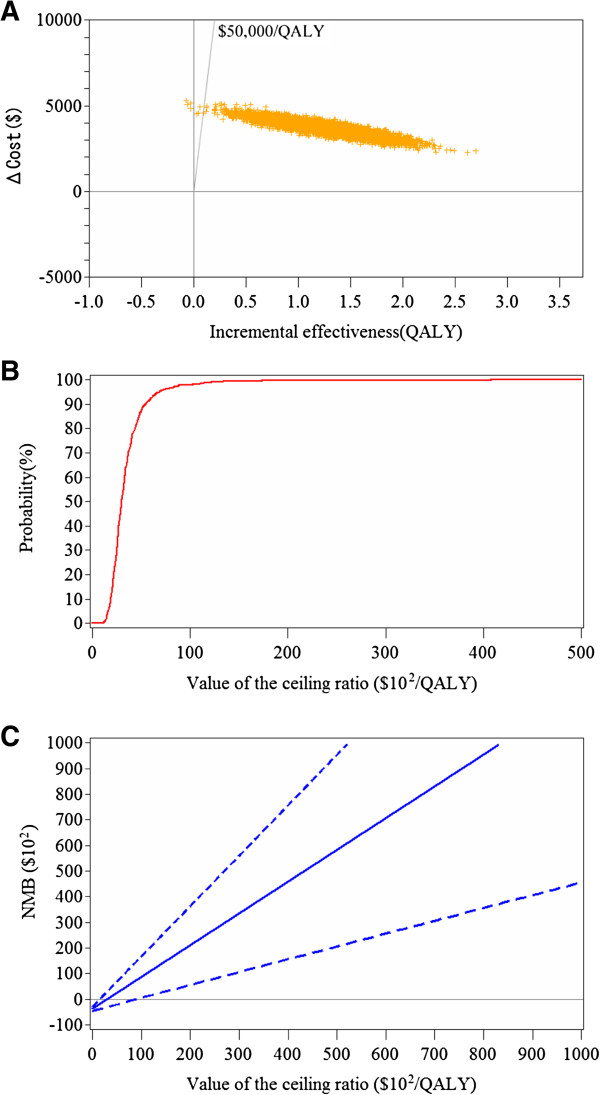
**Stochastic sensitivity analyses. (A)** Incremental cost-effectiveness scatter plot of adjuvant S-1 therapy, **(B)** Cost-effectiveness acceptability curve of adjuvant S-1 therapy, **(C)** Net monetary benefit curve of adjuvant S-1 therapy with 95% confidence intervals.

The results of probabilistic sensitivity analyses are shown in Figures 2. Figure 2A shows ICER (cost per QALY gained) scatter plots based on 5,000 samples. All points resided in the northeast quadrant (i.e., more effective and more costly). All points were located under the diagonal line indicating the ICER of $50,000 per QALY gained. The CEAC is presented in Figure 2B. If the value of an additional QALY was $6,220, the likelihood of S-1 therapy being cost-effective was 95%. The NMB curve is shown in Figure 2C. The value of an additional QALY was $3,016, when the NMB curve crossed the horizontal axis.

A number of qualitative sensitivity analyses are shown in Tables 2 and 4. As to time horizon (Table 2), from 5-year observation to over lifetime, ICER varied from $12,716 to $3,016, as mentioned before.

**Table 4:** One-way sensitivity analysis of important factors

**Factor**	**Cost-effectiveness ratio ($/QALY gained)**
Base case analysis	3,016
Simulation model	
Log-normal	2,874
Weibull	3,341
Recurrence rate (95% CI: 30.5% - 38.8%)	2,446 ‒ 3,891
Utility	
Remission after surgery (95% CI: 0.788 - 0.898)	2,825 ‒ 3,231
Metastasis (95% CI: 0.231 - 0.473)	2,998 ‒ 3,032
QALY gained (95% CI: 0.48 – 1.96)	1,901 ‒ 7,696
Recurrence cost (95% CI: $2,032 - $2,422)	2,834 ‒ 3,149
End of life cost (95% CI: $3,997 - $4,766)	2,682 ‒ 3,302
Exclusion of end-of-life costs due to gastric cancer	3,677
S-1 cost (95% CI: $4,322 - $4,772)	2,810 ‒ 3,173
Total cost difference (95% CI: $2,911 - $4,512 )	2,347 ‒ 3,638

Discount rate: 3% for both cost and effectiveness, Period: lifetime.

The two-way sensitivity analysis of discount rate for both costs and effect showed a relatively small change in ICER. ICER was lowest ($2,194/QALY) without discounting and highest ($3,628/QALY) at the discount rate of 5% for both costs and effectiveness. ICER increased with increase in discount rate of both cost and effect.

The results of one-way sensitivity analyses are shown in Table 4. Variations in recurrence rate, utility value, QALYs, the acquisition cost of S-1, recurrence cost, end-of-life cost, and simulation model did not greatly change ICER. With variations of these variables, ICERs varied from $1,901 to $7,696 per QALY gained.

## Discussion

From the perspective of the National Health Insurance in Japan, this cost-effectiveness analysis showed that S-1 adjuvant therapy for gastric cancer gained LYs and QALYs, while it increased costs, compared with surgery alone (Table 2). The ICER of S-1 therapy can be ranked close to the top of the league table of cost-utility in oncology [33]. There is some consensus about the threshold of willingness to pay for additional QALY internationally (e.g., $50,000 in the US, £30,000 in the UK, or AUS $42,000 in Australia) [34]. A recent review suggested that the plausible threshold is $109,000/QALY, rather than $50,000/QALY [35]. In Japan, the social value (i.e., willingness to pay) for QALY gained was estimated to be from $53,000 to $56,000 by a nationwide mail survey using conjoint analysis [36]. Since the ICER of S-1 therapy is far below these thresholds, it is considered acceptable.

There has been little evidence on economic evaluation of adjuvant therapy for gastric cancer. A cost-effectiveness analysis evaluating postoperative chemoradiotherapy for gastric cancer in the US showed that the incremental cost-effectiveness ratio was $38,400 per QALY gained [37]. This ratio is 14 times higher and less efficient than that in our study, although several factors such as clinical practice patterns and relative costs should be considered in transferring evaluation data [14]. Moreover, since there is no genuine utility information in calculating QALY in the report [37], its validity and plausibility would be questionable.

The results of this study are subject to uncertainty and assumptions. To estimate stochastic uncertainty of ICER due to sampling variation or error, probabilistic sensitivity analyses [14,31,32] were performed (Table 2, Figure 2). Cost-effectiveness scatter plots showed that all points of ICERs were located under the diagonal line indicating $50,000/QALY. CEAC and NMB curves give more information. If a decision-maker was willing to pay $6,220 to achieve an additional QALY, the likelihood of S-1 therapy being acceptable as cost-effective was 95% (Figure 2B). The NMB curve shows that S-1 therapy was beneficial, if a decision-maker was willing to pay $2,782 (Figure 2C). These values are extremely low compared with the thresholds (e.g., $50,000).

The time horizon is an important issue to sufficiently capture relevant costs and health outcomes of S-1 adjuvant therapy. The observation period of the ACTS-GC, 5 years was limited. While most costs were incurred mainly in the observational period, LYs gained would continue after it. In this study, a simulation model was used to extrapolate its results. There is a variety of ways for simulation [18], but no uniform methodology available. We used the Boag model, which is indicated to be predictive for prognosis of gastric cancer [17]. In a sensitivity analysis, the ICER of the observational period was much higher than that of over lifetime (the base case), but it is very low compared with the thresholds. Also, the results of other simulation methods indicated similar results. The exclusion of end-of-life costs due to gastric cancer slightly increased the ICER, but it still remained far under the threshold (Table 4). These analyses show the robustness of this study.

The key drivers of cost-effectiveness results of S-1 are mainly the acquisition cost of S-1 and the costs related to recurrence and death. The S-1 therapy partly offset the acquisition cost of S-1 by the savings achieved by reduction of these costs. In one-way sensitivity analysis (Table 4), varying recurrence rates and costs of recurrence and end-of-lie did not have substantial impact on cost-effectiveness. Varying acquisition cost, which was the other cost driver, also did not have major impact on cost-effectiveness (Table 4). The sensitivity analysis of total cost corresponded with these results.

Cost-effectiveness analysis using QALYs offers the opportunity to consider both quantity and quality of survival. However, no substantial difference in ICERs was observed between cost per LY gained and QALY gained (Table 2). In this study, utility values were derived from a relatively small number of patients with gastric cancer, but this is the first study which directly evaluated the utilities among patients with gastric cancer. These values are similar to those observed for general cancer (i.e, 0.89 after surgery and 0.44 for metastasis) in the Canadian survey among the general population [38]. The sensitivity analysis on range of utility values for remission after surgery and metastasis revealed no major change in cost-effectiveness (Table 4). In a sizable fraction of cost-effectiveness analyses, utility weighting was indicated not to substantially alter the estimated cost-effectiveness of an intervention [39]. It is thus suggested that sensitivity analyses using ad hoc adjustment or weight from the literature may be sufficient. Our results support this conclusion.

The impact of discounting for the time value of money on the results was examined extensively by two-way sensitivity analysis. Although ICERs were more sensitive to effectiveness discounting than cost discounting, there was no substantial change in cost-effectiveness. The main reason is likely to be that major costs were incurred during the early phase of follow-up and improved survival continued for a relatively long time.

There are additional limitations in the analysis that should be commented on. First, the perspective of this analysis is that of a payer for healthcare, rather than a society. From a societal perspective, the range of costs is broader and includes other costs such as indirect costs. Since S-1 therapy increased OS and decreased recurrence, these factors would reduce indirect costs and decrease its ICER.

Second, the issue of generalizability of this study to other countries should be carefully examined. S-1 is widely used in Asian countries (e.g., Japan, Korea, Singapore and China). However, it is difficult to determine the relative effectiveness of S-1, compared with the preoperative chemoradiotherapy in the US and the preoperative triplet-chemotherapy in Europe, since there is no direct comparison among them [8-10]. Moreover, there are several critical arguments around these studies. For example, the INT-0116 study attracted some criticism on the grounds of poor standardization of surgery and insufficient extended dissection of regional lymph nodes [5]. Thus it was argued that the chemoradiation component of the adjuvant treatment had compensated for less-than-ideal surgery. On the other hand, the quality of the MAGIC trial was pointed out to be much poorer than that of the INT-0116 study, in the areas of active quality control of surgery, data management, and compliance with protocol [12]. As to S-1, a difference in S-1 phamacokinetics was observed between Asians and Caucasians [13].

Recently, although the subjects did no have resectable gastric cancer like in this study, but advanced gastric cancer, the First-Line Advance Gastric Cancer Study (FLAGS) [40], a multinational trial, showed that cisplatin/S-1 was statistically non-inferior in overall mortality to cisplatin/5-FU and showed a significantly improved safety profile in Western countries. While S-1 is now approved by the EMEA in European countries, an international head-to-head comparison between S-1 therapy and the Western standard therapies will be required to confirm relative effectiveness and cost-effectiveness of S-1 therapy.

## Conclusion

S-1 adjuvant therapy for gastric cancer gained LYs and QALYs, while it increased costs, compared with surgery alone. The ICER of S-1 therapy can be ranked close to the top of the league table of cost-utility in oncology and far below the social value or threshold for QALY gained in Japan. S-1 therapy for curatively resected gastric cancer is likely cost-effective. This therapy can be accepted for wide use in Japan.

## Appendix: the method of QALY calculation

A.1 Calculation of QALY

QALYi (u), defined as the QALY at year i, was calculated by the following Equation (1), in which uNR represents the utility value of no relapse and uR represents the utility value of relapse.

(1) $$\begin{array}{l} \mathrm{QALYi}\left(u\right)=\mathrm{uNR}\times \mathrm{mean}\;\mathrm{relapse}-\mathrm{free}\;\mathrm{rate}+\mathrm{uR}\\ \;\;\;\times \left(\mathrm{mean}\;\mathrm{survival}\;\mathrm{rate}–\mathrm{mean}\;\mathrm{relapse}-\mathrm{free}\;\mathrm{rate}\right)\; \end{array}$$

If d is the discount rate, the equation becomes *QALY(u)=Σid(i-1) × QALYi(u)*.

The mean rate of survival was calculated as the area under the curve (AUC) of OS, and the mean rate of relapse-free survival was calculated as the AUC of RFS, using the trapezoidal approximation rule.

A.2 Estimate of survival curves of lifetime OS

When estimating the survival curves of lifetime OS, it was assumed that some patients in this study would be cured in response to treatment. This model is called the Boag (cure) model or mixture cure model. This statistical model assumes a mixed distribution of survival time among cured patients and uncured patients.

Y is defined as a variable indicating the presence or absence of cure in patients. Y = 0 stands for cure, and Y = 1 stands for non-cure. If p is defined as the probability of non-cure as represented by p = Pr(Y = 1), and T is a random variable indicating the survival time, the cumulative distribution function of T is represented by the following Equation (2).

(2) $$\begin{array}{l} F\left(t\right)=\mathrm{Pr}(T\leq t)\\ \;=p\cdot \mathrm{Pr}\;\left(T\leq t/Y=1\right)+(1-p)\cdot \mathrm{Pr}\;(T\leq t/Y=0)\; \end{array}$$

It was assumed that no events occur because of cure in cured patients. In other words, if Pr(T ≤ t|Y=0) = 0, the distribution function would be represented by Equation (2). This is referred to as a cure model.

(3) $$F\left(t\right)=p\cdot \left(t/Y=1\right)\;$$

In the cure model, the probability density function f(t) and survival function S(t) are represented by the following Equations (4).

(4) $$\begin{array}{l} f\left(t\right)=p\cdot f(t/Y=1)\\ S\left(t\right)=(1-p)+p.S(t/Y=1) \end{array}$$

A logistic regression model was assumed to calculate the probability of non-cure *p*. In this model, *p* is calculated by Equation (5), in which z is a covariance vector, x = (1,z)*'* (*'* stands for vector transposition), and b is a regression coefficient vector of covariance.

(5) $$p\left(x\right)=\frac{\exp\left(b'x\right)}{1+\exp\left(b'x\right)\;}$$

The Boag model [15] assumes a log-normal distribution for the survival time of uncured patients, but a log-logistic distribution was assumed in the present study. Furthermore, sensitivity analysis was also performed assuming a log-normal distribution and a Weibull distribution, and the maximum likelihood method was used to estimate the parameters using observational data of the ACTS-GC trial [10,11]. The goodness of fit of the model was evaluated with Akaike’s information criteria (AIC). A log-logistic distribution has two parameters **θ** = (γ, λ)′, and the survivor function is as follows:

(6) $$S\left(t;\theta \right)=\frac{1}{1+\lambda \cdot t^{\gamma }}$$

The statistical software package SAS (version 9.2) was used to fit the data to the aforementioned models, and the probabilities of non-cure (*p*) were estimated to be 0.306 and 0.422 in the S-1 group and surgery alone group, respectively. The log-logistic distribution parameters λ and γ were 0.9724 and 0.4121, respectively. The value of AIC for the log-logistic model was 1,678. Those for log-normal and Weibull models were 2,113 and 2,117, respectively. The programs used to estimate the model parameters were the SAS macro for survival models with a cured fraction (Mixture Cure Models).

To examine the validity of the log-logistic model, the distribution of survival time of cured patients was also analyzed using data on patients with gastric cancer obtained from the Cancer Institute Hospital (1946–2004), which has an open database [19]. The approach used was as follows: First, data on patients who met the following 6 eligibility criteria corresponding to the ACTS-GC trial (n = 1,457) were extracted from all data (n = 13,740). The median age of the patients extracted from the database was 57 years, which was 6 years younger than the median age of 63 years in the ACTS-GC trial. Kaplan-Meier curves were plotted using the extracted patient data, defining only death from gastric cancer as an event. The curve reached a plateau after about 20 years (corresponding to an age of 77 years). These data were used for cure models assuming a Weibull distribution, log-normal distribution, and log-logistic distribution. The goodness of fit of the data as indicated by the AIC was best for the log-logistic distribution. While the value of AIC for the log-logistic model was 1,845, those for the log-normal and Weibull models were 2,071 and 2,105, respectively.

Eligibility criteria of the ACTS-GC trial

1) A histologically confirmed diagnosis of gastric cancer

2) Lymph-node dissection of D2 or greater, with a curability of A or B

3) Stage II, IIIA, or IIIB disease

4) No liver metastasis, hematogenous metastasis, or distant metastasis

5) An age of 20 to 80 years

6) No previous treatment (chemotherapy, radiotherapy) received

Finally, the OS curve was constructed by combining the disease-specific survival curve (cure parametric model) and the disease-independent survival curve (the general population matched for age and sex of the subjects) based on the competing risk model. The actual calculation was done using a competitive risk model and the following Equation (7), in which S_B_(t) stands for the survival rate in the disease-specific survival curve (= cure model curve), S_C_(t) stands for the survival rate of the general population in the disease-independent survival curve, and S_A_(t) is the estimated rate of OS after the observation period. The structure of the OS curve was presented in Figure 1B.

(7) $$S_{A}\left(t\right)=S_{B}\left(t\right)\;S_{C}\left(t\right)\;$$

## Competing interest

MS reports receiving lectures fees from Taiho. All other authors: none to declare.

## Authors’ contributions

AH: study concept and design, acquisition of economic data, analysis and interpretation of economic data, and preparation of manuscript.MS, SN: acquisition of subjects and/or clinical data, analysis and interpretation of clinical data. All authors read and approved the final manuscript.

## Pre-publication history

The pre-publication history for this paper can be accessed here:

http://www.biomedcentral.com/1471-2407/13/443/prepub
